# Clinical and radiographics results at 3 years of RCT with split-mouth design of submerged vs. nonsubmerged single laser-microgrooved implants in posterior areas

**DOI:** 10.1186/s40729-019-0196-0

**Published:** 2019-12-18

**Authors:** Renzo Guarnieri, Dario Di Nardo, Gianni Di Giorgio, Gabriele Miccoli, Luca Testarelli

**Affiliations:** 1 Department of Dental and Maxillofacial Sciences, School of Dentistry, University La Sapienza, Rome, Italy; 2 Treviso, Italy

**Keywords:** Submerged two-stage, Nonsubmerged one-stage, Dental implants, Marginal bone loss

## Abstract

**Aim:**

To evaluate and compare radiographic crestal bone loss (CBL) and soft tissue parameters around submerged/two-stage and nonsubmerged/one-stage single implants with the same endosseous portion (body design and surface, thread design and distance) and identical intramucosal laser-microgrooved surface, after 3 years of loading.

**Materials and methods:**

Twenty submerged/two-stage implants and 20 nonsubmerged/one-stage implants were placed randomly with a split-mouth design in the posterior areas of 20 partially edentulous patients.

Radiographic and clinical examinations were carried out at the implant placement, at the delivery of prosthetic restorations, and at each year of the follow-up period. Plaque index (PI), probing depth (PD), bleeding on probing (BOP), and gingival recession (REC) were recorded. Radiographic crestal bone levels were assessed at the mesial and distal aspect of the implant sites. In addition, the influence of the vertical keratinized tissue thickness (KTT) on CBL was investigated.

**Results:**

At the delivery of prosthetic restorations, a statistically significant difference (*P* = 0.013) was found in radiographic mean CBL between submerged and nonsubmerged implants (0.15 ± 0.05 mm vs. 0.11 ± 0.04 mm). At the end of the follow-up period, no statistical difference (*P* = 0.741) was found in the mean CBL between submerged and nonsubmerged implants (0.27 ± 04 mm vs. 0.26 ± 0.5 mm). The changes in the soft tissues including PI, PD, BOP, and REC had no significant differences in either group. Moreover, KTT did not show a statistical correlation with CBL.

**Conclusions:**

After 3 years of loading, no statistical difference was noted in CBL and soft tissue conditions between single submerged two-stage and nonsubmerged one-stage laser-microgrooved implants.

**Trial registration:**

http://clinicaltrials.gov/ct2/show/NCT03674762

## Introduction

In the last decades, the replacement of missing teeth with implant-supported restorations has become a predictable treatment with excellent long-term results [1]. It is based on the concept of intimate interfacial contact between the bone and functionally loaded dental implants, defined as “osseointegration” by Brånemark et al. [2, 3] and “functional ankylosis or direct bone apposition to the titanium surface” by Schroeder et al. [4]. According to Branemark et al.’s and Schroeder et al.’s clinical guidelines, two main implant designs, two-piece/submerged and one-piece/nonsubmerged, and two surgical protocols, two-stage/one-stage, have been developed. In the two-stage surgical approach, the top of the implant is placed at the level of the alveolar crest, and abutment connection is performed 3 to 6 months later, during a second surgery. In a one-stage approach, the top of the implant is placed above the bone crest, leaving the implant collar to protrude through the soft tissue. Thus, it does not require a second surgery for abutment connection. Many studies have demonstrated comparable outcomes with both implant designs and surgical approaches [5–11]. Based on current clinical recommendations, the one-stage approach might be preferable to shorten treatment times, while a two-stage submerged approach could be indicated when the implant is not expected to obtain optimal primary stability or in association with GBR [12]. Moreover, comparative studies between the two surgical protocols have highlighted other advantages for non-submerged implants, such as the lack of an interface/microgap between the implant and abutment at or below the alveolar crest level, a more mature soft tissue healing due to the lack of a second-stage surgery, and a smaller crown-to-implant ratio [12]. However, in many of these studies, the two-piece submerged and one-piece nonsubmerged implants were not similar in terms of shape, surface characteristics, height of the implant collar, size, component fit, etc. Furthermore, few clinical studies have been published comparing the two different surgical approaches in the same patient [10, 11]. More robust evidence is still needed to determine whether the two different surgical approaches provide the same satisfactory outcomes over time using implants with the same body design and surface, same thread design and pitch, and identical intramucosal surface. Therefore, the aim of this randomized clinical trial was to evaluate and compare radiographic crestal bone loss (CBL) and soft tissue parameters, using a one-stage vs. two-stage surgical protocols, around single submerged and nonsubmerged implants with the same tapered body design and surface, the same thread design and distance, and identical intramucosal surface (laser-microgrooved), placed in a separate section of the posterior mandible or maxilla of the same patient, after 3 years of loading.

## Materials and methods

### Patients

This randomized clinical trial included 20 patients, 12 males and 8 females, between the age of 36 and 64 (mean age of 49.7 ± 12.3 years), who were partially edentulous and needed implants for rehabilitation with a single tooth/implant of two non-adjacent sites. Patients were consecutively enrolled between January and July 2014. The study was approved by the Institutional Ethics committee of La Sapienza University, Rome, Italy, (#4597), and was conducted according to the principles outlined in the Helsinki declaration for biomedical research involving human subjects. Clinical trial registration at http://clinicaltrials.gov/ct2/show/NCT03674762

Inclusion criteria were age ≥ 18 years, good general health, and without contraindications to implant surgery. Exclusion criteria were implants placed into regenerated bone or with grafting/regenerative procedures, lack of a periodontal chart and periapical radiograph at the beginning and at the end of the follow-up period, alcohol and drug abuse, pregnancy, or uncontrolled metabolic disorders, tobacco smoking (> 10 cigarettes/day), full mouth plaque score (FMPS), and full mouth bleeding score (FMBS) ≥ 25%, periodontally compromised patients (with attachment loss ≥ 3 mm and/or radiographic bone loss ≥ 30% of root length in ≥ 30% of sites), teeth adjacent mesially and distally to the implant area affected by untreated periodontal, and/or endodontic infections.

### Implants

Two implants were used:

1. Tapered Internal Laser-Lok® implant (BioHorizons, Birmingham, AL, USA) with laser microgrooved in the range of 8 μm intramucosal design, 3.8 mm and 4.6 mm in diameter and length between 9.0 and 12.0 mm (Fig. 1).

**Fig. 1:**
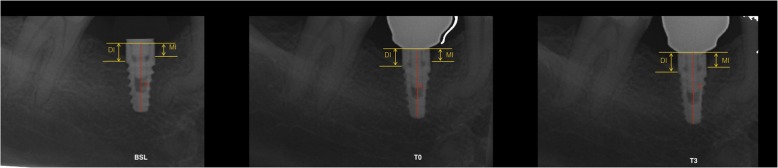
Example of the location of a non-submerged implant, bone, and adjacent tooth
2. Tapered Tissue Level Laser-Lok® implant (BioHorizons, Birmingham, AL, USA) with a laser microgrooved in the range of 8 μm intramucosal design and a 1.3 mm machined metal collar, 3.8 mm and 4.6 mm in diameter, and length between 9.0 and 12.0 mm (Fig. 2).

**Fig. 2:**
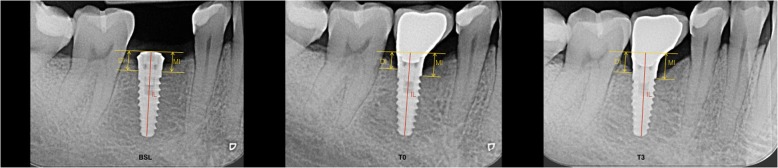
Example of the location of a submerged implant, bone, and adjacent tooth

**Fig. 3:**
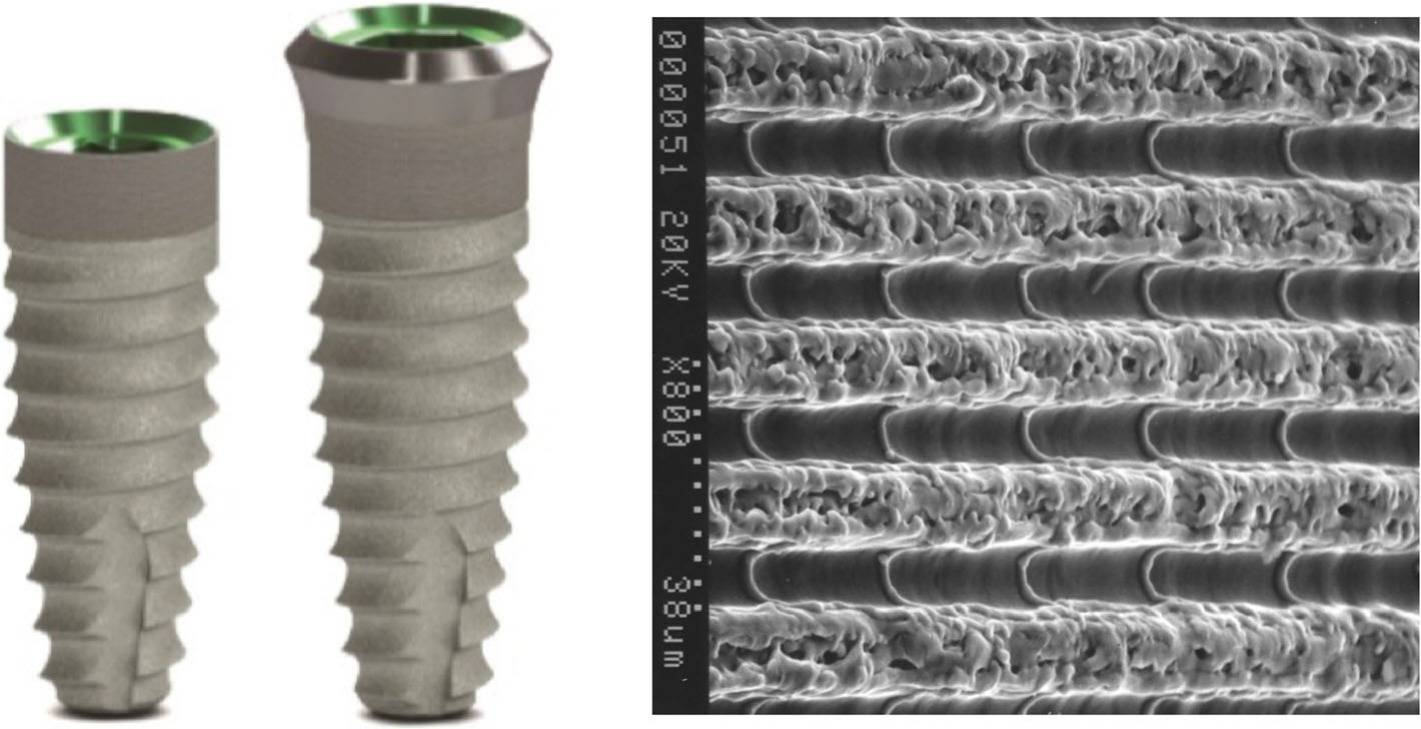
Implants used in the present study and laser-microtextured intramucosal surface (original magnification × 800)

Both implants had the same body tapered macro design, the same resorbable blast textured surface, and buttress thread. Laser-produced microgrooves are a series of cell-sized parallel, linear, and circumferential isotropic channels (Fig. 3 on the right).

The cases were randomly divided into two groups as two-stage/submerged and one-stage/nonsubmerged. Thus, in each patient, the two implants (submerged and nonsubmerged) were placed randomly in the left and right posterior area of the mandible, or in the left and right posterior area of the maxilla (Tables 1 and 2).

**Table 1:** Demographic data of patients, implants position, and type of implant

No. of patients/age (years)/sex	Position	Submerged	Nonsubmerged	Length/diameter (mm)
1/44y/M	14	X		10.5 × 3.8
	26		X	9 × 3.8
2/51y/M	36	X		9 × 4.6
	44		X	9 × 3.8
3/59y/F	35		X	10.5 × 3.8
	46	X		10.5 × 4.6
4/38y/F	47	X		9 × 4.6
	36		X	9 × 4.6
5/57y/M	24		X	12 × 3.8
	15	X		12 × 3.8
6/44y/F	16		X	9 × 4.6
	24	X		12 × 3.8
7/60y/M	36	X		10.5 × 4.6
	46		X	10.5 × 4.6
8/49y/F	15		X	12 × 3.8
	24	X		10.5 × 3.8
9/46y/M	37	X		9 × 4.6
	45		X	9 × 3.8
10/63y/M	25	X		12 × 3.8
	16		X	9 × 4.6
11/55y/M	15	X		10.5 × 3.8
	24		X	10.5 × 3.8
12/45y/F	44		X	9 × 3.8
	36	X		9 × 3.8
13/37y/M	25		X	10.5 × 3.8
	16	X		9 × 4.6
14/53y/F	47	X		9 × 4.6
	37		X	9 × 4.6
15/48y/F	25	X		10.5 × 3.8
	14		X	10.5 × 3.8
16/50y/M	26	X		9 × 3.8
	15		X	10.5 × 3.8
17/34y/M	46	X		9 × 4.6
	36		X	9 × 4.6
18/44y/M	15		X	12 × 3.8
	26	X		9 × 3.8
19/40y/M	34		X	10.5 × 4.6
	46	X		10.5 × 4.6
20/46/F	25	X		10.5 × 3.8
	16		X	9 × 3.8

**Table 2:** Distribution of each implant in each group

Position	Total implants	Submerged	Non-submerged
14	2	1	1
15	5	2	3
16	3	1	2
17	1	-	1
24	4	2	2
25	4	3	1
26	3	2	1
27	0	-	-
34	1	-	1
35	1	-	1
36	5	3	2
37	2	1	1
44	2	-	2
45	1	-	1
46	4	3	1
47	2	2	-

### Pre-surgical assessment

For a complete pre-surgical evaluation, an intra-oral rx and a CBCT scan examination were performed for each implant site.

### Surgical procedures

The surgical procedures were all performed by two operators (RG, LT). Before the implant placement, in each site, the vertical keratinized tissue thickness (KTT) was measured after performing anesthesia, by means of n. 30 K-file inserted until touching the bone crest in the center of future implant placement.

The vertical KTT was dichotomized into two groups (≤ 2 mm and > 2 mm) in accordance with the results of an animal study performed by Berglundh and Lindhe [13]. Implants were placed, with the rough/microgrooved border flush with the bone crest, with the laser-microgrooved surface at the supra crestal level, and at a minimum distance of ≥ 1.5 mm from the adjacent natural teeth.

Patients scheduled for surgery were prescribed systemic amoxicillin/clavulanic acid (Augmentin, GlaxoSmithkline, Italy), 1 g, twice a day for 7 days, and a chlorhexidine digluconate solution 0.12% (Dentosan 0,12%, Johnson & Johnson, USA) rinse (twice daily for 1 min). After local anesthesia by infiltration using articaine/epinephrine (Ecocain 20 mg/ml, Molteni Dental, Italy), surgical access with a midcrestal incision in the center of the edentulous ridge was performed. A minimally extended incision, paramarginal at the adjacent teeth, was released. A full-thickness flap was carried out to expose the crest and the vestibular limit of the bone. Utmost care was taken to preserve the periodontal integrity of adjacent teeth. Following implant placement, the flap was sutured without tension using 4.0 or 5.0 monofilament sutures which were left in place for 10 days. Patients were instructed to have a liquid or semiliquid diet for the first three days and gradually return to a normal diet. An analgesic, ibuprofen 600 mg (Abbott srl, Italy) was prescribed to take immediately after surgery and after 8 h.

In the submerged group, second-stage surgeries for the placement of healing abutments were carried out after 4 months in the mandible and 6 months in the maxilla. This procedure was performed by a midcrestal minimal incision, slightly larger than the coronal diameter of the implant. No secondary surgical manipulation of the soft tissue was performed. Once the healing screw was inserted, suturing was not necessary. Each submerged implant received a titanium healing abutment in height varying from 2 to 4 mm, so as to obtain an overall mucosa emergence of the complex implant/healing abutment, as similar as possible to that of the controlateral non-submerged implant, and in any case not greater than 2 mm.

Prosthetic restorations were delivered after 5 months for implants in the mandible and 7 months for implants in the maxilla. All restorations were screw-retained, and the abutment type was consistent within the same patient, full titanium or hybrid zirconia, depending on the availability of the prosthetic laboratory.

### **Radiographic examination**

Radiographs were taken using a film holder at the time of data collection by means of a long cone technique. For the radiograph procedure, an individualized acrylic resin device was fixed to the residual dentition and a radiograph holder was constructed for each patient. This technique ensured that the same position of the radiograph film could be reproduced at each visit and the angle of the radiograph would not deviate. Radiographs were performed immediately at implant placement (BSL), at the delivery of definitive crowns (T0), and each year after loading (T1, T2, T3). The radiographs were taken in high-resolution mode (Vista Scan Durr Dental, Durr Dental Italy S.r.l, Italy) with a dental x-ray machine (TM 2002 Planmeca Proline CC, Planmeca Group Helsinki, Finland) equipped with a long tube that operated at 70 Kw/7.5 mA. Specialized software (DBSWIN software, Durr Dental Italy S.r.l, Italy) was used for linear measurements of marginal bone changes.

The following radiographic measurements were performed:

- **r**adiographic implant length (IL): distance (in mm) between the implant coronal margin and the implant apex as assessed at the mid portion of the implant
- residual bone height at the mesial (MI) and distal (DI) aspects of the implant: distance (in mm) between the line linking the coronal implant margin and the first contact of the crestal bone on both mesial and distal side of the implant

The radiographic CBL was measured as the difference between MI/DI values at baseline (T0) and at each follow-up examination (T1, T2, T3). For each implant, CBL was calculated as the mean value of MI and DI.

**Fig. 4:**
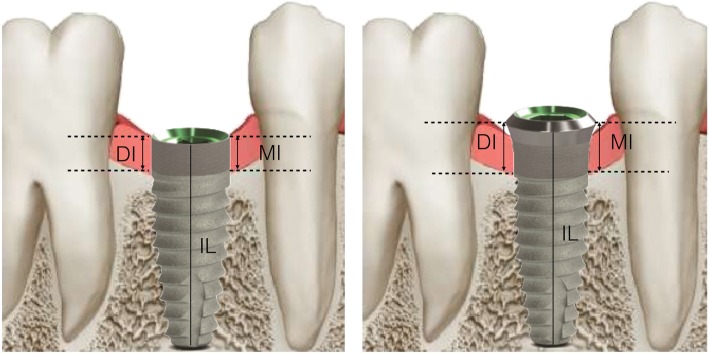
Schematic view of radiographic measurement references

To account for radiographic distortion, radiographic measurements on each radiograph were adjusted for a coefficient derived from the ratio: true length of the implant/IL. All measurements were carried out by a single trained examiner who had previously undergone a calibration session for radiographic assessment on a sample of 5 patients treated with the same implant system and not included in the study (kappa test = 0.9640, SE of kappa = 0.06, 95% confidence interval: from 0.8792 to 1.000). Figures 1 and 2 show an example of radiographic measurements used for evaluation. Figure 4 shows a schematic view of radiographic measurement references.

### Clinical evaluation

Modified plaque index (PI), probing depth (PD), and bleeding on probing (BOP) were recorded at the delivery of definitive restorations (T0) and at each yearly recall visit (T1, T2, T3) on six sites per implant, by means of a manual periodontal probe (PCP UNC-15, Hu Friedy, Chicago, IL, USA). For each implant, the mean of the six measurements was calculated and used for comparison purposes and statistical analysis. Mucosal recession (REC) was recorded at the end of the 3-year follow-up period.

All the clinical outcome variables were carried out by a single trained examiner who had previously undergone a calibration session on a sample of 5 patients treated with the same implant system and not included in the study (kappa test = 0.9418, SE of kappa = 0.09, 95% confidence interval: from 0.8417 to 1.000).

### Statistical analysis

A public domain online software (Raosoft, http://www.raosoft.com/samplesize.html) was used to calculate the minimum number necessary for statistical evaluation. Data were analyzed using SPSS software version 13.0 (Chicago, IL, USA). For clinical parameters (PD and REC) and radiographic CBL, data were calculated for each implant and reported as the mean ± SD, at baseline (T0), at 1-year (T1), 2-year (T2), and 3-year (T3) examination. Number of sites with plaque and number of sites with bleeding at T0, T1, T2, and T3 were also reported. The normality of the distribution of variables was controlled by the Kolmogorov–Smirnov test. The Bonferroni test was used for multiple comparisons between the two groups. The two-factor repeated measure ANOVA was used to compare variables between the groups (submerged and nonsubmerged) at T0, T1, T2, and T3. Parametric test assumptions were not available for PI and BOP; thus, these variables were analyzed with the Wilcoxon signed-rank tests. The results of the Wilcoxon signed-rank tests were expressed as the number of observations (*n*), the mean ± SD. An alpha error of 0.05 was set to accept a statistically significant difference.

## Results

At the end of the follow-up period, no patient dropped off the study, and the survival rate was 100% for both groups of implants.

At the 3-year follow-up, no statistically significant difference was found between the study groups regarding PI and BOP (*P* > 0.05). The number of sites with plaque was 12 (15%) for submerged implants and 11 (13.7%) for the nonsubmerged implants, whereas the mean number of sites with BOP was 14 (17.5%) for submerged implants and 11 (13.7%) for nonsubmerged implants (Table 3). In Table 4 is reported the full mouth index of PPD, Plaque and Bleeding.

**Table 3:** Differences in number of sites with plaque and bleeding on probing (BOP) between the two groups during the follow-up period (Wilcoxon signed-rank tests, *P* > 0.05)

	T0	1-year	2-year	3-year
Number of sites with plaque				
Submerged	7	7	9	12
Nonsubmerged	12	10	8	11
Significance	0.23	0.31	0.22	0.82
Number of sites with BOP				
Submerged	2	10	9	14
Nonsubmerged	6	10	4	11
Significance	0.08	0.75	0.51	0.41

**Table 4:** Patients’ full-mouth periodontal probing depth (FMPPD), full-mouth plaque score (FMPS), and full-mouth bleeding score (FMBS) recorded during the follow-up period

	FMPPD (mm)	FMPS (%)	FMBS (%)
	Mean (SD)	Mean (SD)	Mean (SD)
Baseline	1.6 (0.3)	13.7 (2.1)	11.4 (1.7)
3-year follow-up (T3)	1.8 (0.2)	15.1 (1.4)	12.3 (1.4)
Significance	0.77	0.81	0.39

**Fig. 5:**
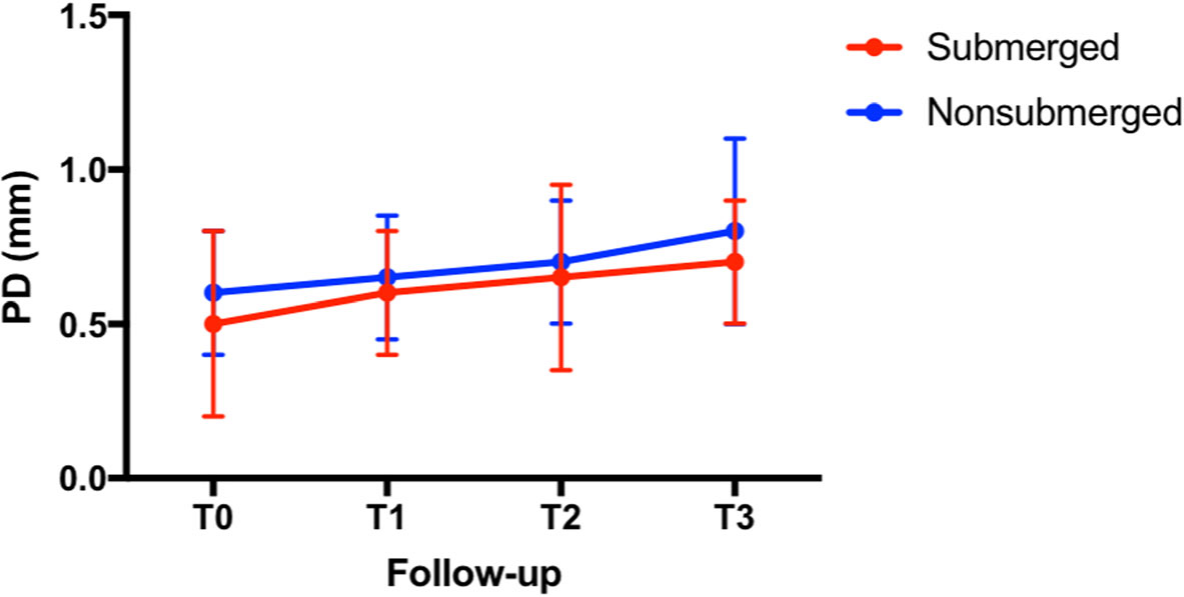
Mean values of probing depth (PD) between the two groups during the follow-up period. ANOVA test *P* > 0.05

Submerged implants had a mean PD value of 0.5 ± 0.3 mm at T0 and 0.7 ± 0.4 mm at T3. No statistically significant difference was noted between T0 and T3 (*P* < 0.05). Nonsubmerged implants had a mean PD value of 0.6 ± 0.2 mm at T0 and 0.8 ± 0.1 at T3. No statistically significant difference was noted between T0 and T3 (*P* > 0.05). No statistically significant difference in PD was noted at T3 between the two groups (*P* > 0.05) (Fig. 5).

**Fig. 6:**
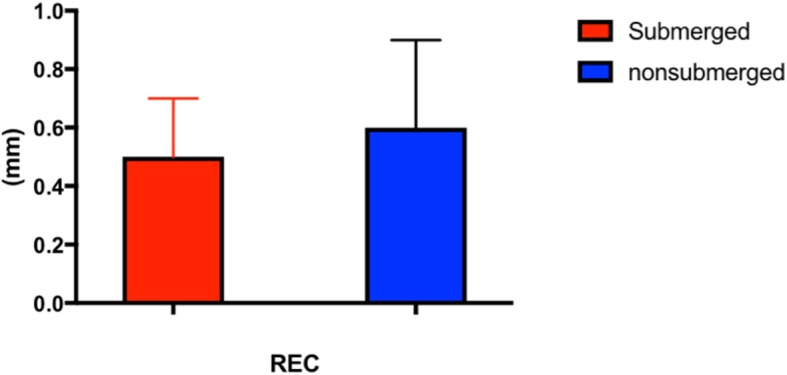
Mean values of gingival recession (REC) between the two groups at the end of follow-up period (3-year). ANOVA test

**Fig. 7:**
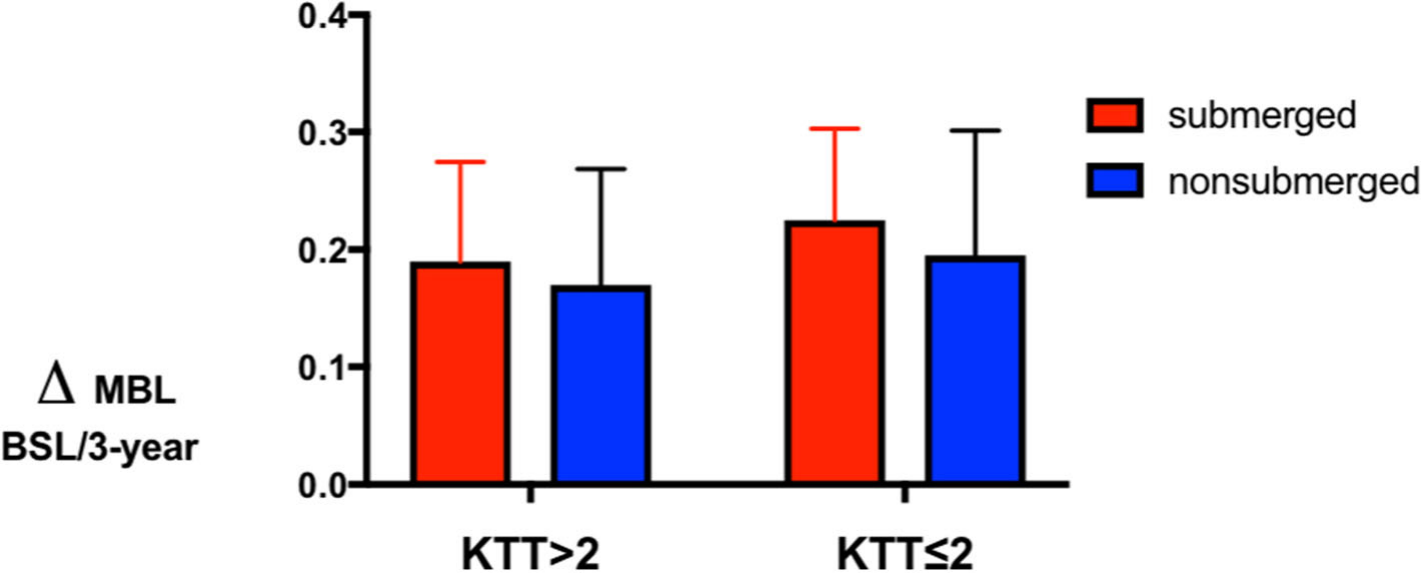
Changes of CBL (mm) between the two groups in sites with KKT > 2 and ≤ 2 mm. ANOVA test

After the 3-year observation time, the mean REC value recorded for submerged implants was 0.5 ± 0.2 mm, while the mean REC value for nonsubmerged implants was 0.6 ± 0.3 mm (Fig. 6). Measurements had no statistically significant difference (*P* > 0.05). No statistically significant difference was found in MBL between sites with vertical KTT > 2 mm and ≤ 2 mm (Fig. 7).

**Fig. 8:**
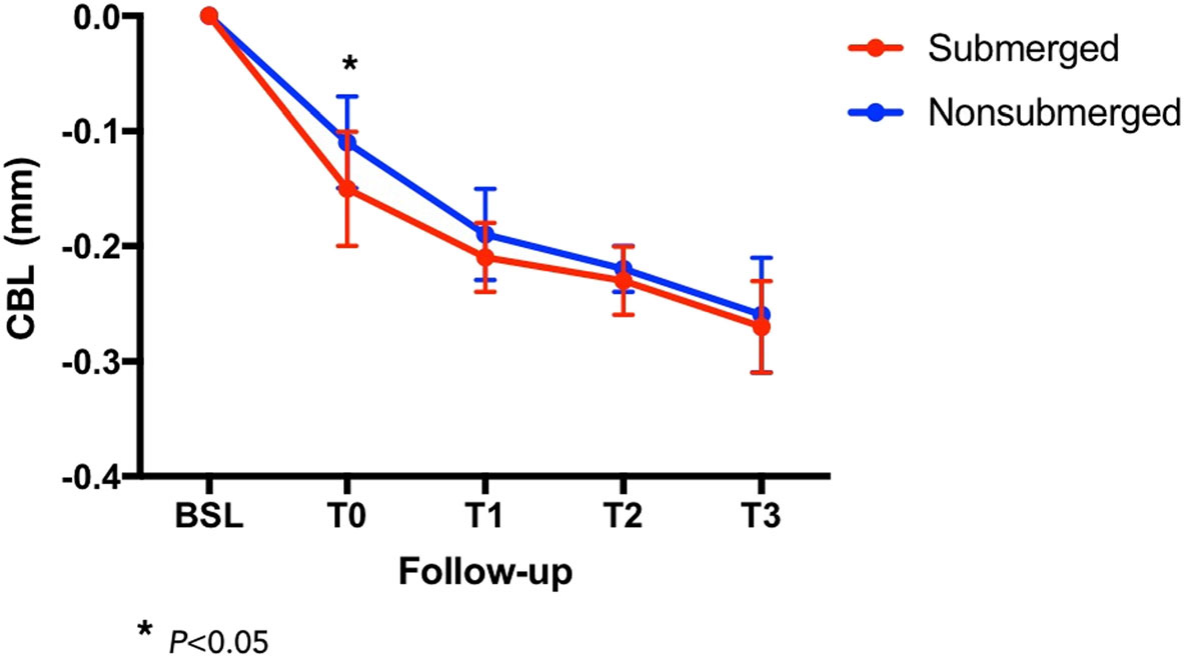
Mean values of crestal bone loss (CBL) between the two groups during the follow-up period. ANOVA test

Radiographic CBL at the delivery of crowns (T0) was significantly greater in submerged implants than that in nonsubmerged (0.15 ± 0.05 mm vs. 0.11 ± 0.07 mm) (*P* < 0.05). At the end of the 3-year follow-up period (T3), submerged implants showed a mean CBL of 0.27 ± 0.6 mm, while submerged implants showed a mean CBL of 0.26 ± 0.7 mm. Difference in mean CBL at T3 between the two groups was not statistically significant (*P* > 0.05) (Fig. 8). Changes of CBL (Δ) between T0 and T3 were not significantly different in the two groups (Δ = 0.12 ± 0.06 mm for submerged implants and 0.15 ± 0.09 mm for nonsubmerged implants).

## Discussion

CBL mean values recorded around submerged and nonsubmerged implants at different timepoints are the most interesting results of the present randomized clinical trial. Before functional loading, radiographic CBL was significantly greater in submerged implants than that in nonsubmerged implants (0.23 mm ± 0.05 mm vs. 0.09 mm ± 0.07 mm). During the follow-up period, both implants showed similar changes in CBL that were not statistically significant (Δ = 0.12 ± 0.06 mm for submerged implants and 0.15 ± 0.09 mm for nonsubmerged implants). These results are in agreement with previously published comparative data between submerged and nonsubmerged implants on the short-term follow-up. A randomized clinical study by Juan Flores-Guillen et al. [14] reported at the end of the five-year follow-up a mean radiographic CBL of 0.59 (SD 0.92) mm and 0.78 (SD 1.03) mm for the submerged and nonsubmerged implants, respectively. Although differences of CBL at the end of the follow-up were not statistically significant, the submerged group demonstrated more crestal bone-level changes (− 0.52 mm versus − 0.24 mm) before functional loading (baseline to 6 months). Moreover, the authors indicated that more than half of the mean CBL reported at 5 years occurred during the healing of the implants and during the establishment of the biological width around the neck of the implant. In that study, however, for calculating the bone-level changes, the date of implant placement was used as a baseline, instead of the placement of the final restoration. Comparative data of peri-implant crestal bone changes between submerged and nonsubmerged implants, using as baseline the placement of the final restoration, were instead reported by Cecchinato et al. [6, 15]. At the end of the 5-year follow-up, the difference in CBL around submerged and nonsubmerged implants was not significant. However, between the baseline and the first year examination, the mean marginal bone loss for submerged implants was 0.17 (SD 0.5) mm versus 0.02 (SD 0.38) for nonsubmerged. Similar results related to CBL around submerged and nonsubmerged implants have been reported also by Siadat et al. [16] and Gheisari et al. [17]. In both studies, before loading, compared with submerged implants, nonsubmerged implants showed less CBL, but after 6 and 12 months of function, no significant differences were noted. A possible explanation of the greater CBL before loading around submerged implants could be related to the histological process of bone repair after the detachment of the periosteum carried out in the second surgical procedure, that in the one-stage surgical protocol is avoided [3]. Another possible explanation is the presence of an interface/micrograp at or below the alveolar crest in the submerged group. Histological analyses in animals have documented that bone-level implants placed in submerged and non-submerged (connected to healing abutments) approaches have a similar amount of bone loss [18]. A physiologic reaction to the presence of a microgap/interface seems to be connected to the microbial contamination at the microgap/interface [19–21], which in turn is associated with a significant inflammatory cell infiltrate [22]. In comparison, the complete absence of such microgap/interface produces no inflammation and consequently, no bone loss. Moreover, the magnitude of inflammation is proportionally dependent on the microgap/interface position relative to the alveolar crest. Subcrestal or crestal implant abutment microgap/interface promoted a significantly greater density of inflammatory reaction correlated with bone loss compared to supracrestal interfaces [22].

Data from available literature indicate that if submerged/nonsubmerged techniques do affect CBL, this effect could be associated with the post-operative healing period [9, 23, 24]. In the present study, at the end of the follow-up period (3 years), no significant difference was detected in CBL around submerged and nonsubmerged implants. A possible explanation for this observation could be that stimuli at the bone-implant interface led to the functional adaptation of the bone and connective tissue to the loading situation and to a similar differentiation, resulting in an equal CBL between submerged and nonsubmerged implants.

Few comparative studies between submerged and nonsubmerged implants reported data on PD. At the end of the 3-year follow-up, Sanz et al. [25] in a randomized controlled clinical trial found a similar mean PD value of 2.5 mm around both implants. Similar values have been reported also by the RCT of Flores-Guillen et al. [14] who, after 5 years of loading, founded a mean PD value of 2.40 (SD 0.7) mm for submerged implants and 2.31 (SD 0.40) mm for nonsubmerged implants. In the present study, after 3 years of function, the mean PD value for submerged implants was 0.7 ± 0.4 mm, while for nonsubmerged implants was 0.8 ± 0.1 mm. Differences in PD, compared with overmentioned RCTs, could be related to the presence of a laser-produced microgrooved collar surface on the investigated implants. Histological results in humans documented that the application of this technology allows to obtain a physical attachment of connective tissues to the microgrooved collar. The high mechanical stability and functional orientation of the connective fibers may allow the formation of a soft-tissue seal, which counteract the junctional epithelium downgrowth, the peri-implant marginal bone remodeling, and the PD [26]. Based on the study by Nevins et al. [27], in which connective tissue reattachment to the laser-microgrooved surface was documented, it is possible to hypothesize that the same functional peri-implant soft tissue apparatus may have formed around submerged and non-submerged implants with laser-microgrooved surface to protect the underlying bone. This hypothesis is supported also by the fact that, after the initial greater MBL occurred around the submerged implants, probably connected to the second surgery, during the 3 years of function both implants have showed similar changes of MBL (Δ = 0.12 ± 0.06 mm for submerged implants, and 0.15 ± 0.09 mm for nonsubmerged implants).

Few studies evaluated the influence of vertical KTT on CBL at the time of implant placement [28–30]. Linkevicius et al. [30] investigated the influence of vertical KTT on CBL around implants placed 2 mm supracrestally (non-submerged/test) and implants placed at bone level (submerged connected with healing abutments/control), after 1 year of loading. In sites with vertical KTT ≤ 2 mm, all implants underwent additional CBL, regardless of crestal or supracrestal location of the microgap/interface. In sites with vertical KTT > 2 mm, test implants had significantly less CBL compared with control implants. In addition, there was no statistically significant difference between test and control implants with thin tissues. Contradicting the assumption that placement of a microgap/interface above bone level can prevent CBL [31–34], results by Linkevicius et al. showed that crestal bone was maintained only if vertical KTT was > 2 mm.

In the current study, the vertical gingival thickness was measured at the time of surgery in the center of the osteotomy. A mean value of 1.74 ± 0.9 mm was recorded with no statistical difference between sites with vertical KTT > 2 mm and ≤ 2 mm. A possible explanation for the difference in findings compared with Linkevicius et al. could be related to the method used for measuring the vertical KTT. Linkevicius et al. performed the measurements using a periodontal probe after partial flap deflection. However, this method presents possible bias as a result of non-standardized periodontal probe inclination, flap incision line angulation, and flap mobility.

One limitation of the present study may lie in the fact that the sites compared were not the same (for example molar vs. premolar areas). However, each contralateral implant site was in the same arch (mandible or maxilla) and intercalated between mesial and distal teeth with similar hard and soft tissue conditions. Other limitations of the present study include the use of two different abutments (full titanium and hybrid zirconia), the small sample size, and lack of histological data. Therefore, further studies with an increased number of samples, longer follow-up, and histological data on laser-microgrooved submerged vs. non-submerged implants are still necessary to confirm the reported findings.

## Conclusions

After 3 years of loading, no differences were founded in CBL and soft tissue conditions between single submerged two-stage and non-submerged one-stage laser-microgrooved implants.

## Data Availability

The datasets used and analyzed during the current study are available from the corresponding author on reasonable request.

## References

[1] Esposito M, Coulthard P, Thomsen P, Worthington HV (2005). Interventions for replacing missing teeth: different types of dental implants. Cochrane Database Syst Rev..

[2] Brånemark PI, Breine U, Adell R, Hansson BO, Lindstrom J, Ohlsson A (1969). Intraosseous anchorage of dental prostheses. I. Experimental studies. Scand J Plast Reconstr Surg..

[3] Brånemark PI, Hansson BO, Adell R, Breine U, Lindström J, Hallén O (1977). Osseointegrated implants in the treatment of the edentulous jaw. Scand J Plast Reconstr Surg.

[4] Schroeder A, Pohler O, Sutter F (1976). Tissue reaction to an implant of a titanium hollow cylinder with a titanium surface spray layer. SSO Schweiz Monatsschr Zahnheilkd.

[5] Buser D, Mericske-Stern R, Bernard JP (1997). Long-term evaluation of non-submerged ITI implants. Part 1: 8-year life table analysis of a prospective multi-center study with 2359 implants. Clin Oral Impl Res.

[6] Cecchinato D, Olsson C, Lindhe J (2004). Submerged or non-submerged healing of endosseous implants to be used in the rehabilitation of partially dentate patients. J Clin Periodont.

[7] Becktor JP, Isaksson S, Billström C (2007). A prospective multicenter study using two different surgical approaches in the mandible with turned Brånemark implants: conventional loading using fixed prostheses. Clin Impl Dent Rel Res.

[8] Cordaro L, Torsello F, Roccuzzo M (2009). Clinical outcome of submerged vs. non-submerged implants placed in fresh extraction sockets. Clin Oral Impl Res.

[9] Moustafa Ali RM, Alqutaibi AY, El-Din Gomaa AS, Abdallah MF (2018). Effect of submerged vs nonsubmerged implant placement protocols on implant failure and marginal bone loss: a systematic review and meta-analysis. Int J Prosthodont.

[10] Ericsson I, Randow K, Nilner K, Petersson A (1997). Some clinical and radiographical features of submerged and non-submerged titanium implants. A 5-year follow-up study. Clin Oral Implants Res.

[11] Astrand P, Engquist B, Anzén B (2002). Nonsubmerged and sub-merged implants in the treatment of the partially edentulous maxilla. Clin Implant Dent Relat Res.

[12] Esposito M, Grusovin MG, Chew YS, Coulthard P, Worthington HV (2009). One-stage versus two-stage implant placement. A Cochrane systematic review of randomised controlled clinical trials. Eur J Oral Implantol.

[13] Berglundh T, Lindhe J (1996). Dimension of the periimplant mucosa. Biological width revisited. J Clin Periodontol.

[14] Flores-Guillen J, Álvarez-Novoa C, Barbieri G, Martín C, Sanz M (2018). Five-year outcomes of a randomized clinical trial comparing bone-level implants with either submerged or transmucosal healing. J Clin Periodontol..

[15] Cecchinato D, Bengazi F, Blasi G, Botticelli D, Cardarelli I, Gualini F (2008). Bone level alterations at implants placed in the posterior segments of the dentition: outcome of submerged/non-submerged healing. A 5-year multicenter, randomized, controlled clinical trial. Clin Oral Impl Res.

[16] Siadat H, Panjnoosh M, Alikhasi M, Alihoseini M, Bassir SH, Rokn AR (2012). Does implant staging choice affect crestal bone loss?. J Oral Maxillofac Surg..

[17] Gheisari R, Eatemadi H, Alavian A (2017). Comparison of the marginal bone loss in one-stage versus two-stage implant surgery. J Dent (Shiraz)..

[18] Hermann, J.S, Cochran, D.L., Nummikoski, P.V. & Buser, D. Crestal bone changes around titanium implants. A radiographic evaluation of unloaded non-submerged and submerged implants in the canine mandible. J Periodontol 1997; 68: 1117–1130. 10.1902/jop.1997.68.11.1117 9407406

[19] Quirynen M, van Steenberghe D (1993). Bacterial colonization of the internal part of two-stage implants. An in vivo study. Clin Oral Impl Res.

[20] Persson LG, Lekholm U, Leonhardt Å, Dahlen G, Lindhe J (1996). Bacterial colonization on internal surfaces of Brånemark system^A^ implant components. Clin Oral Impl Res.

[21] Jansen VK, Conrads G, Richter E-J (1997). Microbial leakage and marginal fit of the implant–abutment interface. Int J Oral Maxillofacial Impl.

[22] Broggini N, McManus LM, Hermann JS, Medina RU, Oates TW, Schenk RK (2003). Persistent acute inflammation at the implant-abutment interface. J Dent Res.

[23] Abrahamsson I, Berglundh T (2009). Effects of different implant surfaces and designs on marginal bone-level alterations: a systematic review. Clin Oral Imp Res.

[24] Al Amri MD (2016). Crestal bone loss around submerged and nonsubmerged dental implants: a systematic review. J Prosthet Dent.

[25] Sanz M, Ivanoff CJ, Weingart D, Wiltfang J, Gahlert M, Cordaro L, Ganeles J, Bragger U, Jackowski J, Martin WC, Jung RE, Chen S, Hammerle C (2015). Clinical and radiologic outcomes after submerged and transmucosal implant placement with two-piece implants in the anterior maxilla and mandible: 3-year results of a randomized controlled clinical trial. Clin Implant Dent Relat Res..

[26] Nevins M, Nevins ML, Camelo M, Boyesen JL, Kim DM (2008). Human histologic evidence of a connective tissue attachment to a dental implant. International Int J Periodontics Restorative Dent.

[27] Nevins M, Camelo M, Nevins ML, Schupbach P, Kim DM (2012). Reattachment of connective tissue fibers to a laser-microgrooved abutment surface. Int J Periodontics Restorative Dent.

[28] Suárez-López Del Amo F, Lin GH, Monje A, Galindo-Moreno P, Wang H-L (2016). Influence of soft tissue thickness on peri-implant marginal bone loss: a systematic review and meta-analysis. J Periodontol.

[29] Akcali A, Trullenque-Eriksson A, Sun C, Petrie A, Nibali L, Donos N (2017). What is the effect of soft tissue thickness on crestal bone loss around dental implants? A systematic review. Clin Oral Impl Res.

[30] Linkevicius T, Apse P, Grybauskas S, Puisys A (2009). The influence of soft tissue thickness on crestal bone changes around implants: a 1-year prospective controlled clinical trial. Int J Oral & Maxillofacial Impl.

[31] Hermann JS, Buser D, Schenk RK, Cochran DL (2000). (2000) Crestal bone changes around titanium implants. A histometric evaluation of unloaded non-submerged and submerged implants in the canine mandible. J Periodontol.

[32] HermannJ S, Buser D, Schenk RK, Schoolfield JD, Cochran DL (2001). Biologic width around one- and two-piece titanium implants. Clinl Oral Impl Res.

[33] Hermann JS, Schoolfield JD, Schenk RK, Buser D, Cochran DL (2001). Influence of the size of the microgap on crestal bone changes around titanium implants. A histometric evaluation of unloaded non-submerged implants in the canine mandible. J Periodontol.

[34] Derks J, Håkansson J, Wennström JL, Tomasi C, Larsson M, Berglundh T (2015). Effectiveness of implant therapy analyzed in a Swedish population: early and late implant loss. J Dent Res.

